# Exploring the Use of *Bryophyllum* as Natural Source of Bioactive Compounds with Antioxidant Activity to Prevent Lipid Oxidation of Fish Oil-In-Water Emulsions

**DOI:** 10.3390/plants9081012

**Published:** 2020-08-11

**Authors:** Pascual García-Pérez, Sonia Losada-Barreiro, Carlos Bravo-Díaz, Pedro P. Gallego

**Affiliations:** 1 Applied Plant & Soil Biology, Plant Biology and Soil Science Department, Biology Faculty, University of Vigo, 36310 Vigo, Spain; pgallego@uvigo.es; 2 CITACA, Agri-Food Research and Transfer Cluster, University of Vigo, 32004 Ourense, Spain; 3 Physical Chemistry Department, Chemistry Faculty, University of Vigo, 36310 Vigo, Spain; cbravo@uvigo.es; 4 REQUIMTE-LAQV, Chemistry and Biochemistry Department, Science Faculty, University of Porto, 4169-007 Porto, Portugal

**Keywords:** *Kalanchoe*, plant tissue culture, polyphenols, PUFAs, omega-3, fish oil emulsions, medicinal plants, plant biotechnology

## Abstract

The current industrial requirements for food naturalness are forcing the development of new strategies to achieve the production of healthier foods by replacing the use of synthetic additives with bioactive compounds from natural sources. Here, we investigate the use of plant tissue culture as a biotechnological solution to produce plant-derived bioactive compounds with antioxidant activity and their application to protect fish oil-in-water emulsions against lipid peroxidation. The total phenolic content of *Bryophyllum* plant extracts ranges from 3.4 to 5.9 mM, expressed as gallic acid equivalents (GAE). The addition of *Bryophyllum* extracts to 4:6 fish oil-in-water emulsions results in a sharp (eight-fold) increase in the antioxidant efficiency due to the incorporation of polyphenols to the interfacial region. In the emulsions, the antioxidant efficiency of extracts increased linearly with concentration and levelled off at 500 μM GAE, reaching a plateau region. The antioxidant efficiency increases modestly (12%) upon increasing the pH from 3.0 to 5.0, while an increase in temperature from 10 to 30 °C causes a six-fold decrease in the antioxidant efficiency. Overall, results show that *Bryophyllum* plant-derived extracts are promising sources of bioactive compounds with antioxidant activity that can be eventually be used to control lipid oxidation in food emulsions containing (poly)unsaturated fatty acids.

## 1. Introduction

The aging of world population is a global trend with important implications in the human health and in the incidence of age-related diseases. Thus, the occurrence of multifactorial diseases, such as cardiovascular, neurodegenerative and neoplastic diseases, has a common trait, represented by the onset of oxidative stress [1]. As a solution, some life-style patterns, including diet and exercise, have become increasingly important in stimulating molecular mechanisms in attempting to prevent the onset of oxidative stress via reactive oxygen and nitrogen species (ROS/RNS) formation [2]. Because of the health implications, in the last decades, the increasing consumers’ awareness about food processing and food quality pushed the food industry to meet new requirements in preparing healthier foods. This way, innovative biotechnological strategies have been developed to make possible the manufacturing of food products with high nutritional added-value and longer shelf-lives [3].

**Scheme 1:**
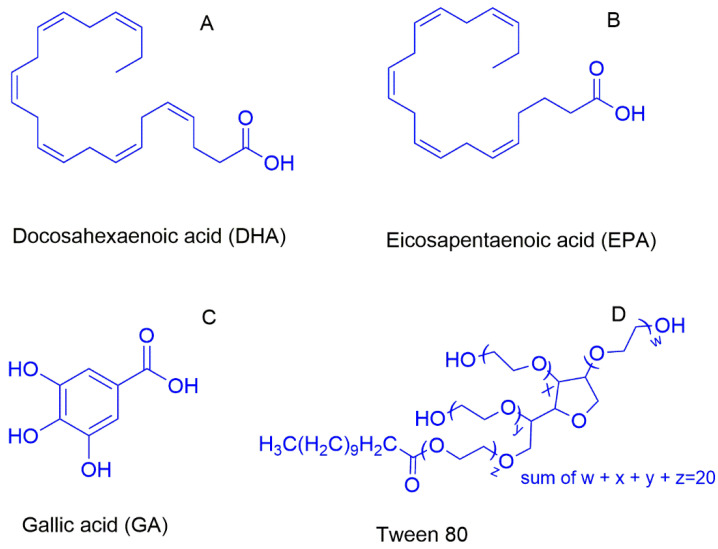
Chemical structures of some of the polyunsaturated fatty acids, (**A**) docosahexaenoic acid (DHA) and (**B**) eicosapentaenoic acid (EPA) present in the fish oil employed in this work. (**C**) The antioxidant gallic acid (GA), used as antioxidant reference standard, and (**D**) the surfactant Tween 80 employed in the preparation of the oil-in-water emulsions.

Lipids are one of the main food components because of their crucial role in human health and nutrition [4]. In addition, the consumption of saturated fatty acids (SFAs) has been linked to a high risk of different cardiovascular diseases and diabetes, and food agencies worldwide recommend a significant reduction of SFAs in foods [5]. SFAs have been preferentially used in foods thanks to their high oxidative stability compared to that of lipids with a variable degree of unsaturation (polyunsaturated fatty acids, PUFAs). Thus, the addition of PUFAs (Scheme 1), one of the major constituents of fish and vegetal oils, has arisen as a precious alternative for enriching food products with bioactive lipids due to their health-enhancing properties [6,7].

**Scheme 2:**
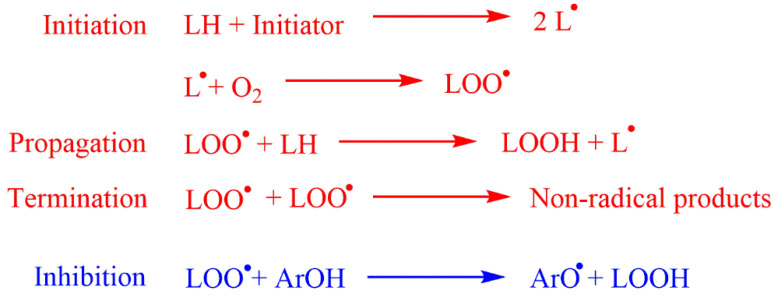
Relevant reactions developed during free radical oxidation (red) of lipids (LH) and its inhibition (blue) by phenolic (ArOH) antioxidants. L^●^ = lipid radical, LOO^●^ = peroxyl radical, LOOH = hydroperoxide, ArO^●^ = phenolic antioxidant radical.

However, the use of PUFAs in food matrices is not exempt from difficulties, since they show an extremely high sensitivity to oxidation (Scheme 2), resulting in a severe loss of food quality driven by the accumulation of oxidation harmful by-products, such as aldehydes and ketones [8,9]. In parallel, as demonstrated in biological systems, lipids are one of cell constituents showing a high sensitivity to ROS and RNS during the development of oxidative stress, thus forming deleterious lipid oxidized derivatives that cause a severe impairment of cell physiology [10]. The lipid oxidation reaction is a complex radical process, usually described in terms of the initiation, propagation and termination steps (Scheme 2). In the initiation step, an initiator (as it could be the case of ROS and RNS) abstracts an allylic hydrogen atom from a polyunsaturated fatty acid (LH), yielding a carbon-centered lipid radical (L^●^) that reacts very rapidly with molecular oxygen to form a lipid peroxyl radical (LOO^●^). In the propagation step, the LOO^●^ peroxyl radicals may abstract an H-atom from another LH molecule (rate-determining step of the propagation reaction) giving a new lipid radical (L^●^) and a lipid hydroperoxide (LOOH). This reaction undergoes various cycles to form a variety of potentially harmful products until the reaction stops when two radicals react to yield a non-radical product (termination step).

PUFAs are usually present in food matrices as a lipid phase dispersed in an aqueous solution coated by a surfactant or emulsifier, that creates a thin interfacial layer separating the oil and water, i.e., they are present as oil-in-water (O/W) emulsions [11]. Emulsions are thermodynamically unstable systems that undergo spontaneous phase separation, but can be stabilized kinetically for some time by employing surfactants, which are adsorbed at the oil-water interface creating a coat around dispersed droplets that prevents phase separation [12]. At the interface, the surfactant molecules point their hydrophilic moieties towards the aqueous phase, whereas their hydrophobic parts are mostly located in the oil phase.

**Scheme 3:**
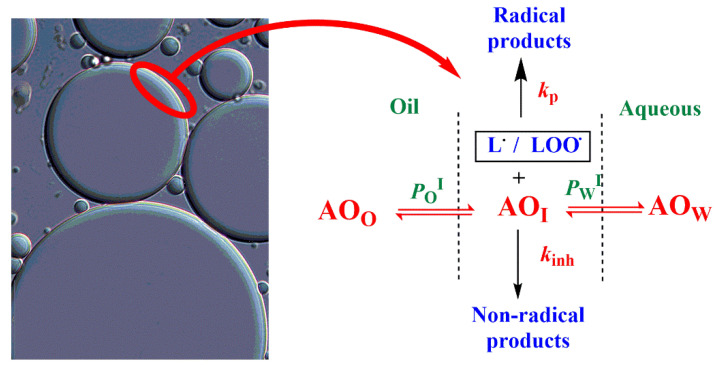
Left. Optical microscopy photograph of oil droplets in an O/W emulsion. Right. Two-dimension representation of a small volume from an O/W emulsion containing the oil, interfacial and aqueous regions. The representation also shows the reactions of radicals, L^●^ and LOO^●^, with a lipid molecule, LH (propagation rate constant, *k*_p_), and with an antioxidant, AO, that partitions between the oil-interfacial (*P*_O_^I^) and aqueous-interfacial (*P*_W_^I^) regions of the O/W emulsion (inhibition rate constant, *k*_inh_). Propagation and inhibition reactions are displayed in Scheme 2.

Consequently, food emulsions are chemically unstable because their containing PUFAs present in the emulsion may be exposed to oxygen, light and/or radiation and may suffer oxidation (Scheme 2). The initiation reaction is likely to initiate in the oil core or in the interfacial region of the emulsion droplets (a potential initiation in the aqueous phase is not probable, since lipids are water-insoluble). According to the floating peroxyl radical theory [13,14], peroxyl radicals (formed in the oil region) are quite polar and diffuse towards the interfacial region of the emulsions, which is more polar than oil, where they are solvated by water (Scheme 3).

To minimize the effects derived from the oxidation of PUFAs, several approaches such as oxygen removal, light protection, and low temperatures are currently employed in food processing, manipulation, and storage. Nevertheless, these strategies have a limited applicability as they are expensive, sometimes difficult to implement and their efficiency depends strongly on additional factors, including the nature of food matrix. A much more practical and cost-effective approach exploits the use of antioxidants, such as phenols (ArOH, Scheme 2), which are molecules capable of efficiently quenching the peroxyl radicals, LOO^●^, by regenerating the parent lipid LH and forming a much less reactive radical, ArO^●^. Details on the reactions and on their rate constants can be found elsewhere [13,14].

The oxidative stability of the emulsion depends, therefore, on the relative rates of radical formation (propagation step in Scheme 2) and antioxidant-mediated inhibition (Scheme 2). An antioxidant, AO, is effective when the rate of its reaction with the peroxyl radicals, r_inh_ = *k*_inh_[ArOH][LOO^●^], is equal to, or faster than the rate of the propagation reaction (r_p_ = *k*_p_[LH][ LOO^●^]) [15]. The efficiency of AOs depends on the chemical nature of the antioxidant (number and position of –OH groups) [16,17] and on their interfacial concentration [18,19,20,21]. Consequently, AOs should be targeted to the interfacial region of the emulsions and increase in as much as possible its effective concentration [18,20,21]. Otherwise, the propagation rate could eventually exceed antioxidant inhibition (r_inh_ < r_p_), thus resulting in a loss of antioxidant efficiency coupled to chemical emulsion instability.

Among the great variety of foods containing a high proportion of PUFAs, fish oils constitute a largely exploited food product, as they are considered a natural source presenting an important nutritional value and health benefits on a variety of cardiovascular, cancer and mood disorders [6,7]. Their use has increased notably in the last years as energy source in parenteral nutrition and pharmaceutical formulations, although they are especially susceptible to oxidation. Fish oils contain a high PUFA proportion, including eicosapentaenoic acid, EPA, and docosahexaenoic acid, DHA (Scheme 1). Both PUFAs present a high degree of unsaturation in their chemical structures, making fish oils especially prone to oxidation, thus requiring an effective protection from oxidative degradation to maintain their nutritional value [4].

In this sense, artificial antioxidants (AOs) are being gradually replaced with natural ones obtained from plant sources to satisfy the current demands of consumers for food naturalness [22,23], and the food industry is facing a continuous search of novel approaches to successfully add natural AOs with nutritional and health benefits to food matrices. Plants, as a result of their secondary metabolism, synthesize a plethora of metabolites with associated bioactivities, as it is the case of polyphenols [24]. Thus, plant-based polyphenols have been widely reported as antioxidants, with the ability of attenuating ROS- or RNS-induced oxidative damage. Therefore, plant bioactive compounds have been used from ancient times as natural sources of bioactive compounds for the treatment of multiple diseases and in therapeutic applications [25,26,27]. They have also been successfully added to food matrices to prevent their oxidative spoilage under different storage conditions [23,25,26] and, because of their health benefits, to prepare functionalized preparations with high added-value. Nevertheless, as secondary metabolites, they are found in limited concentrations in plant tissues and efficient strategies should be developed in order to increase their production [28].

Plant tissue culture (PTC) emerged as a solid biotechnological tool to increase the production of efficient natural antioxidants (polyphenols) with high nutritional and/or therapeutic value [29,30]. PTC offers some advantages in terms of the production of natural antioxidants, since it enables a significant improvement of the rather limited production and yields associated to conventional plant breeding [28]. To investigate the plant-derived antioxidant efficiency in controlling the oxidative stability of fish-oil emulsions, we employed PTC as a tool for their production by three medicinal species belonging to subgenus *Bryophyllum* (genus *Kalanchoe*). *Bryophyllum* species are widely used worldwide in traditional medicine (for instance, in Africa, Asia and South America) for the treatment of several diseases [31]. Their health benefits are commonly attributed to their ability to synthetize antioxidants capable of controlling oxidative stress in human cells [31].

In this work, our main objective was to obtain an efficient and sustainable biological system for the production of polyphenolic compounds [24,31] and to analyze the efficiency of their derived plant extracts to minimize the oxidation process associated with fish oil-in-water emulsions. To simulate different storage conditions, different factors including the effects of plant extract concentration, the oil-to-water ratio used in the preparation of the emulsion, the acidity of the aqueous phase and the effects of temperature, were evaluated. For the sake of comparisons, we used gallic acid (GA, Scheme 1) as the reference AO because GA is one of the most potent natural AOs present in numerous plant extracts, including *Bryophyllum* species [32]. We hope that the results of the proposed set of experiments will help to promote the use of natural plant-derived products as antioxidants and in the development of novel omega-3 containing foods with high nutritional added value.

## 2. Results and Discussion

### 2.1. Total Phenolic Content and Antiradical Activity against DPPH^•^ of Bryophyllum Extracts

Table 1 shows the total phenolic content (TPC, expressed as total gallic acid equivalents in mmol per liter of emulsion, GAE) obtained for the different *Bryophyllum* species. TPC values varied significantly from 3.43 mM GAE (BT) to 5.91 mM GAE (BH). These results are similar to those reported in previous works, obtained for these species cultured in vitro under the same extraction conditions [30], and present a sharp increase in their concentrations in comparison to aqueous BH extracts (0.17 mM GAE) [32]. Previous phytochemical analyses conducted on *Bryophyllum* spp. allowed to identify a variety of phenolic acids, including gallic, ferulic, and caffeic acids, and flavonol derivatives, such as kaempferol glycosides in BD extracts [33,34], and quercetin and flavone glycosides, gallic acid, and some of its derivatives in BT extracts [35,36]. These results are in line with the preliminary identification experiments performed on *Bryophyllum* extracts by HPLC-DAD-ESI/MS^n^, as extracts were constituted by phenolic acids and flavonols, mainly represented by kaempferol, myricetin, and quercetin, and anthocyanins, mostly malvidin derivatives (Supplementary Table S1). It is important to note that flavonols and anthocyanins from *Bryophyllum* plant extracts are present as glycosylated forms, which represents a significant physiological feature that modulates solubility and the antioxidant activity associated to these compounds in plant tissues [37]. These findings indicate that *Bryophyllum* plant extracts contain a complex (heterogeneous) mixture of polyphenols, together with other families of bioactive compounds, which can exert a moderate antioxidant activity, as it is the case of bufadienolides [38,39]. Such compound heterogeneity found in the extracts make difficult the attribution of the antioxidant activity to discrete compounds. Thus, in this work we determined the antioxidant activity of full extracts, because mixtures of individual antioxidants may show synergistic or antagonistic effects, making them impossible to quantify [40]. Evidence of these effects was already pointed out by other authors, showing both synergistic and antagonistic effects against polyphenols in multicomponent systems, mostly due to the regeneration mechanisms that occur between antioxidants, through the formation of stable intermolecular complexes [41,42]. The conditions selected for performing the extraction of phenolic compounds were previously assessed using artificial intelligence-based tools, with the aim of predicting the critical factors affecting this process [30]. In this sense, 80% MeOH was predicted as the most efficient solvent for phenolic extraction from aerial parts from *Bryophyllum* spp. cultured in vitro. In addition, solvent was removed after the concentration step of the extracts, and the solid residue was resuspended in citrate buffer, with the aim of avoiding the use of organic solvent on food-related matrices, as it was the case of fish oil emulsions [43].

The antiradical activity (IC50) of TPCs in the three *Bryophyllum* species tested is different, as indicated in Table 1, following the order BH (239.6 µM GAE) > BD (259.7 µM GAE) ~ BT (259.5 µM GAE). For comparative purposes, Table 1 also shows that the antiradical activity of GA, which is significantly lower (IC50 = 163.4 µM) than that of *Bryophyllum* extracts. The efficiency of antiradical activity of *Bryophyllum* extracts relies on the IC50 values obtained for other medicinal plants, ranging from 35.3 µM for *Thea chinensis* [44] to 28 mM for *Eryngium viviparum* [29]. Furthermore, the antiradical activity of extracts from in vitro-cultured *Bryophyllum* extracts was substantially higher, as IC50 values ranging from 361.5 to 8564.5 µM were obtained for *Bryophyllum* species from conventional breeding [34], thus suggesting that the introduction of *Bryophyllum* under in vitro conditions induces the biosynthesis of secondary metabolites with antioxidant activity.

These findings support the evidence that the antiradical activity of plant extracts strongly depends on different factors, such as the genotype and solvent used for the extraction [30]. In this sense, the antiradical activity of *Bryophyllum* extracts has been maximized by performing the in vitro culture using culture media formulations with deficient macronutrient concentrations, accompanied by the selection of 55–85% aqueous MeOH as the best solvent to perform the extraction of phenolic compounds from aerial parts. In fact, solvent solubility was shown to be the most significant factor that influences antioxidant activity on in vitro-cultured *Bryophyllum*-derived extracts [30].

### 2.2. Antioxidant Efficiency of Bryophyllum Extracts in Fish Oil-In-Water Emulsions

To get some insights into the hydrophilic-hydrophobic character of polyphenols found in *Bryophyllum* extracts, we firstly determined the overall partition constant *P*_W_^O^ between fish oil and water in a binary system (with no added surfactant), thus revealing their behavior in both binary oil-water systems and food emulsions (Scheme 3). Partitioning in multiphasic systems is more complex than in binary mixtures and requires the definition of new distribution constants, since a new region is created upon addition of surfactants to kinetically stabilize the emulsions: the interface [45,46,47]. In this case, distribution is described by two partition coefficients: *P*_O_^I^, between oil and interface, and *P*_w_^I^, between aqueous and interfacial regions. The ratio between *P*_O_^I^ and *P*_w_^I^ is equal to the partition coefficient in the absence of surfactant, *P*_W_^O^ [45,46,47]. *P*_W_^O^ values have, therefore, a great importance to predict the efficiency and distribution of natural antioxidants at different levels of biological organization from binary oil-water systems to living cells [48].

Unfortunately, *P*_W_^O^ values cannot, in general, be predicted from the *P*_W_^OCT^ (octanol-water partition constant) values because partition coefficient values are determined by all intermolecular interactions (electrostatic, hydrogen bonding, and dispersion forces) between the solute and the two phases in which it is dissolved. Its value, thus, depends on the balance of all intermolecular forces involving a solute and the two phases between it is partitioned [49,50,51], making it necessary to determine them for each oil and antioxidant or set of antioxidants [47].

The partitioning experiments were carried out by employing the shake-flask method [52] and showed that most of the antioxidants (>70%) from *Bryophyllum* extracts are located in the aqueous phase. The results contrast with those obtained for GA, which is basically oil-insoluble and >99% of GA [18] remains in the aqueous phase at T = 25 °C. Therefore, the partitioning results allowed us to infer that *Bryophyllum* plant extracts contain polyphenols with a lower polarity than GA. Hence, we can expect those antioxidants to distribute between the oil, interfacial, and aqueous regions when included in emulsions. This is an important issue because hydrophilic antioxidants, such as GA, only distribute between the aqueous-interfacial region and its efficiency in inhibiting lipid oxidation is lower compared to less hydrophilic compounds such as propyl gallate that accumulate in the interfacial region making itself much more effective [18].

It is noteworthy that antioxidant partition constants between oil-interfacial and aqueous-interfacial regions are independent of the oil-to-water ratio employed in the emulsion preparation [18,19,20]. However, the efficiency of antioxidants on emulsions depends on their effective interfacial concentrations, which in turn depends on several factors such as oil and water volumes and surfactant nature [18,19,20,21,53]. Thus, we investigated the effects of the oil-to-water ratio on the efficiency of *Bryophyllum* plant extracts by monitoring conjugate diene (CD) formation along time.

**Figure 1:**
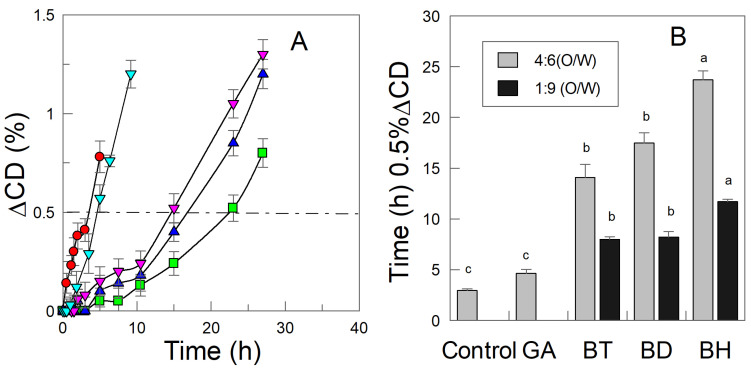
(**A**) Oxidative stability in terms of the variation in CD formation in 4:6 (O/W) stripped fish oil-in-water emulsions in the presence of extracts from different *Bryophyllum* species (■- BH, ▲- BD and ▼- BT, TPC = 500 μM GAE) and ▼- GA ( 500 μM) at T = 25 °C, Φ_I_ = 6.68%. Control (-●-) samples did not contain any antioxidants. The dashed line marks the hours required for a 0.5%- increase in CDs. (**B**) Effect of different *Bryophyllum* extracts on the time required to increase CDs content to 0.5% at two different O/W ratios: 1:9 and 4:6. Data were expressed as average ± standard deviation and different letters indicate significant differences (α = 0.001).

Figure 1A shows a typical kinetic plot obtained for CDs formation in 4:6 (O/W) fish oil-in-water emulsions in the presence of the extracts of three *Bryophyllum* species and in the presence of GA at T = 25 °C. The efficiency of *Bryophyllum* extracts to inhibit lipid oxidation was evaluated, as in previous works [18,19,20,21,53], by determining the elapsed time to increase 0.5% the content of CDs (dashed line in Figure 1A). The values are displayed in Figure 1B and, overall, show that BH extracts were quite effective in delaying CDs formation, showing a higher significant activity than BT and BD extracts (for the same TPC value). The time required to reach 0.5% ΔCD was ~3 h for the control emulsion (no added GA nor plant extracts) and 5 h for GA-loaded emulsions (approximately two-fold). In the case of *Bryophyllum* extracts, the time required increased to ~14 h for BT (4.7-fold), ~17 h for BD (5.7-fold), and ~24 h for BH (eight-fold) in 4:6 (O/W) emulsions. Results, thus, show that the efficiency of *Bryophyllum* extracts is higher than that of GA, following the order BH > BD > BT > GA (Figure 1B). Figure 1B also shows that, in 1:9 (O/W) emulsions, the same efficiency order was reported. It is worthwhile noting that the efficiency order obtained for 4:6 (O/W) emulsions was the opposite to that found in homogeneous systems between GA and *Bryophyllum* extracts (Table 1).

This observation may be rationalized in terms of the effects of antioxidant compartmentalization and oil-to-water ratio on the effective concentration of antioxidants in the interfacial region. Nevertheless, it is physically impossible to isolate the interfacial regions, and thus to identify (by analytical methods) which compounds are located in this region and their effective concentration. Both hydrophobic and hydrophilic antioxidants accumulate in the interfacial region of the emulsions, increasing their effective concentration in the region compared to the stoichiometric one because the interfacial volume is only a small fraction of the emulsion volume [15], but the effective concentration of each antioxidant is different because it depends on the particular partition constants. Since the highest oxidative stability was found for 4:6 emulsions, we only employed these emulsions for subsequent studies.

### 2.3. Effects of Bryophyllum Extract Concentration on Their Antioxidant Efficiency In Fish Oil-In-Water Emulsions

It is now well-recognized the crucial role of the interfacial region in controlling the oxidative stability of emulsions [18,53,54]. We recently demonstrated that there is a direct relationship between the effective interfacial antioxidant concentration and its efficiency: the higher the interfacial antioxidant concentration, the higher its efficiency in inhibiting lipid oxidation [15].

However, the capacity of the interfacial region on solubilizing antioxidants is currently unknown, i.e., we do not know the maximum antioxidant concentration in the interfacial region. This limitation comes, in part, due to the permeability of interfacial region, as it constitutes a highly anisotropic region that makes its isolation physically impossible without perturbing the existing equilibria and where molecules are getting in and getting out at rates close to the diffusion control [55]. Thus, solubility experiments cannot be performed in the system. Molecules residing at the interfacial region experience, in general, a micropolarity that is intermediate between water and oil and we can expect the AO solubility (and any solute located in this region) to depend on the particular composition of the aqueous region, on the nature of the organic solvent employed and, probably most important, on the micropolarity sensed by the antioxidants.

**Figure 2:**
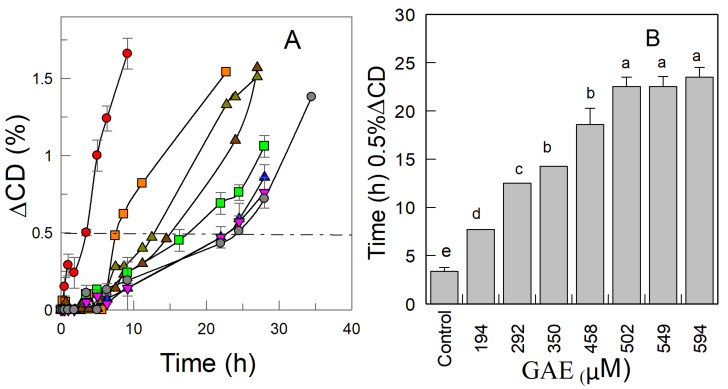
(**A**) Effects of the BH *Bryophyllum* extract concentrations on the variation in CDs formation with time in 4:6 (O/W) stripped fish oil-in-water emulsions. The control samples did not contain any antioxidant (●- control, ■- BH 194 μM, ▲- BH 292 μM, ▲- BH 350 μM, ■- BH 458 μM, ▲- BH 502 μM, ▼- BH 549 μM, ●- BH 594 μM). The dashed line marks the hours required for an increase of 0.5% in CDs formation. (**B**) Effects of amount of BH *Bryophyllum* extract concentration (expressed as μM GAE) on the time required to reach a 0.5% increase in CDs production. Experimental conditions: T = 25 °C, Φ_I_ = 6.68%. Data were expressed as average ± standard deviation and different letters indicate significant differences (α = 0.001).

The use of *Bryophyllum* extracts provides a meaningful opportunity to shed some light on the solubility capacity of the interfacial region of emulsions and for the purpose we evaluated the effects of *Bryophyllum* extract concentrations (TPC values from 194 μM to 594 μM GAE) on the oxidative stability of fish oil emulsions. Figure 2A shows the variations of CDs formation, ΔCD, with time in emulsions loaded with different BH *Bryophyllum* extract concentrations, which were similar to those previously obtained (Figure 1A). The relative antioxidant efficiency was determined by plotting the time required to reach an increase of 0.5% in CDs formation (Figure 2B). Two different behaviors can be distinguished. At low TPC, the required time (0.5% ΔCD) increased linearly upon increasing the amount of TPC until a plateau region was reached at TPC values higher than ~ 500 μM GAE, where an increase in the TPC values had no significant effect on the time (0.5% ΔCD). Similar saturation curves (not shown for the sake of clarity) were found for BT and BD extracts, reaching the plateau region at similar TPC values.

In previous works [18,19,20,21,53], we demonstrated that the reaction between antioxidants and peroxyl radicals mainly takes place in the interfacial region of oil-in-water emulsions. Additionally, it was observed that the oxidative stability of the emulsions correlates with the interfacial concentration of antioxidants: the higher the interfacial concentration is, the higher the oxidative stability is. Results in Figure 2B suggest, therefore, that the linear increase in the oxidative stability of emulsions with BH *Bryophyllum* extract concentration was a consequence of the increasing AOs concentration in the interfacial region, and the plateau region was reached after the saturation of the interfacial region. We hypothesize that, based on the partitioning experiment in binary systems, once the saturation of the interfacial region is reached, antioxidants mainly solubilize in the aqueous region and to a lesser extent in the oil region. To prove this hypothesis, further solubilization experiments in bulk water and oil need to be performed. These experiments are completely out of the scope of this work and will be part of future reports.

### 2.4. Effects of the Acidity of the Aqueous Phase on the Antioxidant Efficiency of Bryophyllum Extracts in Fish Oil-In-Water Emulsions

Changes in the acidity of aqueous phase may modify the degree of ionization of phenolic acids and other polyphenols present in *Bryophyllum* extracts, since pKa values of their carboxylic group are in the range of 4–5 while those of the aromatic –OH groups range from 8 to 9 [45]. The solubility of phenolic anions in water is much higher than that of the neutral compounds and, consequently, changes in acidity may change the interfacial concentrations of AOs present in the interfacial region [56,57]. Changes in pH may also alter the metal chelating ability of polyphenols, which may lead to increase in the possibility of promoting lipid oxidation in the aqueous-interfacial region of the emulsions if free metals are present [56].

To get insights into the effects of acidity, which may affect emulsion manipulation and/or storage conditions, we analyzed the effects of acidity (pH = 3–5) on the efficiency of BH *Bryophyllum* extracts (500 μM GAE) on the oxidative stability of fish oil-in-water emulsions. This pH range was selected as it is found within the range of common food products (pH = 4–7) [17].

**Figure 3:**
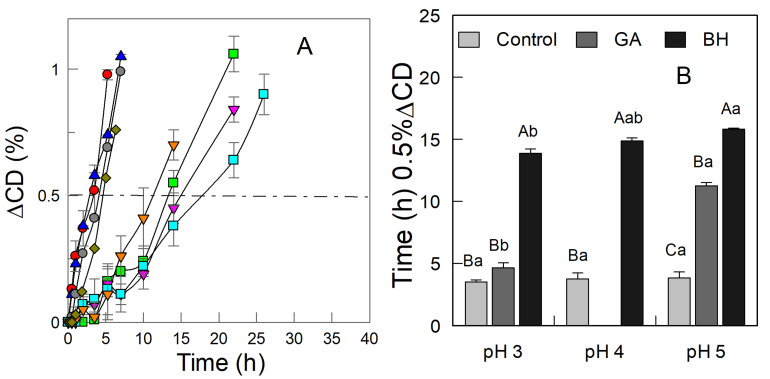
(**A**) Oxidative stability in terms of the variation in CDs formation of 4:6 (O/W) stripped fish oil-in-water emulsions containing BH *Bryophyllum* extracts (TPC 400 μM GAE) and GA (500 μM) at different acidities of aqueous region and at T = 25 °C (Φ_I_ = 6.68%). The control samples did not contain any antioxidant (●- control pH = 3, ▲- control pH = 4, ●- control pH = 5, ▼- GA pH = 5, ◆- GA pH =3, ■- BH pH = 3, ▼- BH pH = 4, ■- BH pH = 5). The dashed line marks the hours required for a 0.5% increase in CDs. (**B**) Effect of the acidity of aqueous region of 4:6 (O/W) emulsions containing BH *Bryophyllum* extracts on the time needed to reach 0.5% ΔCD. Data were expressed as average ± standard deviation. Different lower-case letters indicate significant differences between the same treatments at different pH values and different capital letters indicate significant differences between different treatments within the same pH values (α = 0.001).

Figure 3A displays the variation in CDs formation with time in 4:6 (O/W) emulsions containing BH *Bryophyllum* extracts and GA (included for comparative purposes) at different acidities. Results indicate that in the absence of antioxidants the oxidative stability of emulsions did not show a dependence on the acidity of aqueous region (pH 3–5), but in the presence of BH *Bryophyllum* extract, statistical differences (p < 0.001) were found with increases of 12% at pH = 5 (Figure 3B).

However, the efficiency of GA was 1.4–2.9 times (pH 3–5) lower than that for BH *Bryophyllum* extracts, depending on pH. This behavior can be rationalized in terms of the particular oxidation mechanism of GA: electron-transfer followed by single deprotonation [58]. Cyclic voltammetry studies on the behavior of gallic acid in aqueous buffer solutions show two irreversible waves in the pH range 1.6–6.2 (at higher pHs, the waves are not observed), and upon increasing the pH of the solution, the peak potentials are shifted towards less positive values. Similar results were reported for Trolox in 4:6 (O/W) olive oil-in-water emulsions [56].

### 2.5. Effects of Temperature on the Antioxidant Efficiency of Bryophyllum Extracts In Fish Oil-In-Water Emulsions

**Figure 4:**
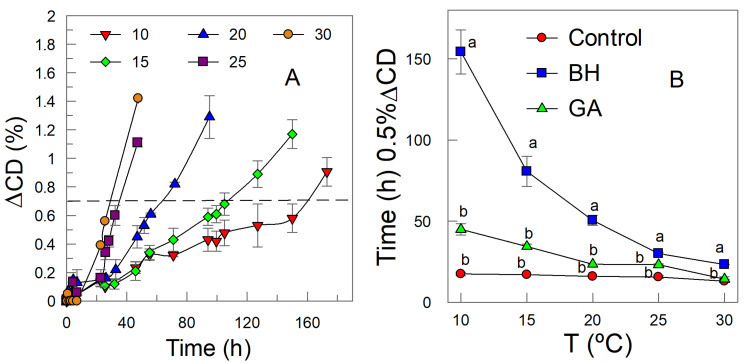
(**A**) Oxidative stability in terms of the variation in CDs formation of 4:6 (O/W) stripped fish oil-in-water emulsions containing BH *Bryophyllum* extracts (TPC 500 μM GAE) at different temperatures (Φ_I_ = 6.68%, pH = 3.0, 0 rpm). The dashed line marks the hours required for a 0.5% CDs increase. (**B**) Effect of the temperature (10 °C–30 °C) in 4:6 (O/W) emulsions containing BH *Bryophyllum* extracts on the time needed to reach 0.5% ΔCD. Data were expressed as average ± standard deviation and different letters indicate significant differences (α = 0.001).

Temperature (T) usually has a paramount effect on antioxidant efficiency because it may strongly affect both the reaction rate constants of antioxidants with ROS and RNS (Scheme 2) and the partition constants (i.e., distribution) of antioxidants between the emulsion regions (Scheme 3). Figure 4A shows the typical kinetic plot for CDs formation, illustrating their formation in 4:6 (O/W) fish oil-in-water emulsions incubated in the absence (control) and in the presence of BH *Bryophyllum* extracts and GA at different temperatures (T = 10–30 °C).

The incubation temperatures ranged from 10 to 30 °C and were chosen to allow emulsions to be oxidized slowly [18] and because they are typical storage conditions of common foods. Figure 4B shows the time required to increase the percentage of primary oxidation products by 0.5% in 4:6 (O/W) emulsions containing BH *Bryophyllum* extracts incubated at different temperatures. In all cases, the oxidative stability of fish oil-in-water emulsions decreased by increasing the temperature (Figure 4B). In the absence of AOs (control), the oxidative stability of emulsions was essentially independent of temperature, with differences less than ~25% upon increasing T from 10 to 30 °C (Figure 4B). At the lowest temperature employed (T = 10 °C), the addition of GA or BH extracts increased the oxidative stability of the emulsions in comparison to the control sample, however, an increase in temperature from T = 10 to 30 °C led to a 6-fold (BH *Bryophyllum* extracts) and two-fold (GA) decrease in the oxidative stability (Figure 4B).

Such decrease in the oxidative stability means that the effects of increasing T on the partition constants prevail over those on the rate constants. In the present case, these two effects work in opposite directions. For one side, according to the Arrhenius equation, an increase in T increases the values of the rate constants of the inhibition reaction (Scheme 2). Nevertheless, at the same time, T increases AOs solubility in the oil and aqueous regions with respect to the interfacial region, thus decreasing their effective interfacial concentrations to a larger extent than the increase in the rate constants [59].

## 3. Materials and Methods 

### 3.1. Chemicals

All chemicals and reagents were of the highest purity available. Gallic acid monohydrate (GA), citric acid, sodium carbonate and 2,2-diphenyl-1-pycrilhydracyl (DPPH^•^) were from Sigma-Aldrich. Tween 80 (E-433) and Folin-Ciocalteu reagent were from Fluka and VWR Chemicals, respectively. All solutions were prepared with deionized water (conductivity < 0.1 mS cm^−1^). The fish oil (Omegatex 3020) was kindly provided by Solutex S.L. (Spain). Fish oil composition (given as % *w*/*w*) was 6.9% SFA, 6.5% oleic acid, 0.54% linolenic acid; and 55.8% PUFAs, 33.8% EPA, and 22% DHA. It was stripped from its naturally present tocopherols by passing twice through a column containing alumina previously activated in an oven at T = 200 °C for 24 h. The stripped fish oil was then stored at T = −20 °C under N_2_ atmosphere to minimize lipid oxidation. The removal of tocopherols was assessed by HPLC, following IUPAC method 2.432 [19]. All glassware was submerged in 37% HCl overnight to remove metals and then rinsed with deionized water.

### 3.2. Plant Material

Three different species from subgenus *Bryophyllum* were used in this work: *Bryophyllum daigremontianum* Raym. – Hamet et Perr. (BD), *Bryophyllum* × *houghtonii* (*B. daigremontianum* × *tubiflorum*, BH) D.B. Ward and *Bryophyllum tubiflorum* Harv. (BT) provided by ADICAM (Spain).

Epiphyllous buds from 18-month-old *Bryophyllum* plants were harvested from a local green-house (42°12′40.0” N 8°43′36.1” W, Vigo, Spain) in December 2018, subjected to the same disinfection protocol reported in previous works [28,32] and cultured in half-macronutrient Murashige and Skoog medium (MS medium, specifically designed for plant tissue culture) [60] supplemented with 3% (*w*/*v*) sucrose and solidified with 0.8% agar (*w*/*v*) at pH = 5.8. Cultures were transferred to a growth chamber at T = 25 °C under a photoperiod of 16 h light and 8 h dark and subcultured every 12 weeks.

### 3.3. Preparation of Bryophyllum Extracts Phenolic Extraction

Experimental conditions for phenolic extraction were previously optimized to maximize the yield from *Bryophyllum* in vitro-cultured plants, as it depends on several factors [30]. In brief, aerial parts from 12-week old *Bryophyllum* plants cultured in vitro were harvested, frozen at T = −20 °C and lyophilized to get a fine powder. One hundred milligrams of dry material were extracted with 10 mL of 80% MeOH (*v*/*v*) and incubated at T = 60 °C for 10 min. Then, samples were cooled down to room temperature and subjected to sonication for 30 min. Later, all samples were centrifuged at 3500 rpm for 10 min. After centrifugation, supernatants were collected and mixed with hexane (1:1) to remove hydrophobic constituents, concentrated by rotary evaporator (T = 40 °C), and resuspended in citrate buffer (pH = 3.0, unless otherwise stated) at a final concentration of 20 mg mL^−1^. Finally, extracts were filtered using PTFE syringe filters (0.22 µm pore size) and stored under N_2_ atmosphere at T = −20 °C until use.

### 3.4. Fish Oil-In-Water Emulsion Preparation

Oil-in-water emulsions were prepared at a final volume of 5 mL by mixing 40% (*v*/*v*) stripped fish oil and 60% (*v*/*v*) aqueous phase, in the case of 4:6 O/W emulsions, and 10% (*v*/*v*) stripped fish oil and 90% (*v*/*v*) aqueous phase, for 1:9 O/W emulsions. In both cases, a fixed amount of emulsifier Tween 80 (Φ_I_ = V_surfactant_/V_emulsion_ = 0.067, Scheme 1) was used. The acidity of aqueous phase was adjusted using citric acid/ sodium citrate buffer to pH = 3.0, unless otherwise stated. Tween 80 was dissolved in the aqueous solution and an aliquot of the aqueous plant extracts was added so that the final concentration of the extract, expressed as total gallic acid equivalents in μmol per liter of emulsion (GAE), was 500 μM GAE, unless otherwise stated. The mixture was then stirred at 20,000 rpm for 1 min at room temperature by using a high-speed rotor (Polytron PT 1600 E) to produce the oil-in-water emulsions. Emulsions without added extract were used as control and emulsions containing only GA were used as a reference standard.

Emulsions were introduced in 25-mL screw–capped glass Erlenmeyer flasks and placed in a thermostated orbital shaker (Incubator Heidolph 1000 orbital stirrer equipped with a Heidolph thermostat 1010 to control temperature) in the dark at constant temperature (T = 10–30 °C, 195 rpm). All experiments were carried out in triplicate and no visual phase separation was observed in any of the emulsions during the time course of the oxidation experiments.

### 3.5. Total Phenolic Determination

The total phenolic content (TPC) of *Bryophyllum* extracts was determined as in previous works [28,30], by employing the colorimetric method reported by Ainsworth and Gillespie [61]. Briefly, 100 µL of plant extracts were mixed with 200 µL of 10% (*v*/*v*) Folin-Ciocalteu’s reagent in water and let stand for 2 min at room temperature. Then, 800 µL of 0.7 M sodium carbonate solution were added. The mixture was incubated for 2 h at room temperature in the dark and the absorbance was measured at λ = 765 nm. TPC was determined spectrophotometrically by interpolation in the previously prepared calibration curve for gallic acid and expressed as total gallic acid equivalents in μM (GAE). The original extracts were diluted with citric buffer solution to fit within the standard curve. All measurements were carried out in triplicate.

### 3.6. Antiradical Activity In Homogeneous Systems: DPPH^•^ Assay

The DPPH^●^ radical scavenging assay has been widely used in several studies to evaluate free radical scavenging activities of plant extracts [23]. The antiradical activity of GA and *Bryophyllum* extracts was determined by the method described by Thaipong et al. [62], based on the reduction of DPPH^●^ radical in the presence of a hydrogen-donating antioxidant. Briefly, 150 µL of extracts were added to 2850 µL of a 110 µM DPPH^●^ methanolic solution. The mixture was vortexed and incubated for 24 h in the dark at room temperature. GA was included as a positive control at the same concentration as the extract solution. The decrease in the absorbance of DPPH^●^ was measured at λ= 517 nm. All measurements were carried out in triplicate and results are expressed as IC50 values, in µM, defined as the concentration of plant extract or GA required to decrease the initial concentration of DPPH^∙^ radical by 50%.

### 3.7. Antioxidant Efficiency In O/W Emulsions: Schaal Oven Test

The Schaal oven test constitutes a simple and reliable method for monitoring the primary products derived from lipid oxidation in emulsified systems. The formation of conjugated dienes (CDs) was monitored by UV-Vis spectroscopy according to the AOCS official method Ti 1a 64 [17].

(1) $$\%CD=1.0769\times \frac{A\left(233nm\right)}{\left[oil\right]}$$

In brief, aliquots (10 µL) of the emulsions were withdrawn at selected times and diluted to 10 mL with ethanol, producing a homogeneous solution whose absorbance was measured at λ = 233 nm [18]. The variation in CD formation with time was obtained and the relative oxidative stability of the different emulsions was assessed as the time (h) required to achieve an increase in CDs formation (ΔCD) of 0.5% in the emulsions, once the propagation step has been reached, i.e., after the lag period. The value of 0.5% ΔCD was chosen, as in previous works [18,19,20,21,53], as a measure of the oxidative stability at early stages to reduce in as much as possible the formation of secondary oxidation products. However, as we showed, similar results are obtained when monitoring the formation of CDs up to ~1% ΔCD (see results section). We have shown in previous works [18,63,64,65,66] that monitoring primary and secondary products under similar conditions to those used here lead to the same conclusions. Likewise, preliminary experiments using fish oil emulsions were performed in order to assure that the variation of CDs correlate with the peroxide content of samples (data not shown). Therefore, the determination of oxidation values according to the AOCS official standard method provides reliable results.

### 3.8. Determination of the Distribution of Antioxidants in Binary O/W Systems

To get a feeling on relative amounts of hydrophobic and hydrophilic antioxidants present in the *Bryophyllum* extracts, we determined their distribution in binary oil-water mixtures. For the sake of comparisons, we also determined the distribution of gallic acid, which was chosen as AO of reference. The distributions were determined, as in previous works, by employing a shake-flask method at T = 25 °C [18,19]. Both 500 µM GAE *Bryophyllum* extracts and 500 µM GA were dissolved, independently, in 6 mL of an aqueous acid buffered (citric/citrate, pH = 3.0) solution and mixed with 4 mL of stripped fish oil. The mixtures were stirred with a high-speed rotor for 1 min. Samples were then transferred to test tubes, allowed to reach thermal equilibrium, and phases were separated by centrifugation for at least 45 min.

The fraction of antioxidants from *Bryophyllum* extracts in the aqueous phase, expressed as μM GAE, was determined as described earlier. The percentage in oil phase was calculated, as usually, by difference between the fraction of added AOs (100%) and that in the aqueous phase, %AO_O_ = 100%AO_W_. All measurements were carried out in triplicate.

### 3.9. Statistical Analysis

One-way analysis of variance (ANOVA), followed by post hoc Tukey HSD test was performed to detect significant differences (α = 0.001) between treatments, using the STATISTICA v. 14 (StatSoft, Inc.) software. In all cases, values are expressed as average ± standard deviation.

## 4. Conclusions

In this work, we proposed the opportunity of employing in vitro-cultured *Bryophyllum*-derived plant extracts, particularly *Bryophyllum × houghtonii* extracts, as a promising source of bioactive compounds with antioxidant activity for minimizing lipid oxidation of omega-3 enriched emulsions. Our results indicated that the antioxidant efficiency of plant extracts was driven by the partitioning of their constitutive polyphenols in the interface, oil, and water regions of fish oil-in-water emulsions. Additionally, it was demonstrated that such efficiency depends on a series of physicochemical factors, such as extract concentration, emulsion composition, pH, and temperature. In this sense, the efficiency of *Bryophyllum* plant extracts on the inhibition of fish oil-in-water emulsions was enhanced under certain experimental conditions: (i) oil-to-water ratio, showing the higher efficiency for 4:6 (O/W) emulsions; (ii) extract polyphenolic concentration, which achieved a maximum value of ~500 μM GAE; (iii) the acidity of the aqueous phase, that caused a 12% increase in antioxidant activity from pH 3.0 to 5.0; and (iv) temperature, which played a significant role in the enhancement of plant extract antioxidant activity of six-fold from 30 to 10 °C.

The characterization of the main factors which affect the antioxidant efficiency of *Bryophyllum* plant extracts is crucial to define effective strategies for controlling and preventing lipid oxidation of omega-3 enriched emulsified systems. Once the efficacy of *Bryophyllum* plant extracts was assessed, future reports will seek to identify the individual compounds with enhanced activity and analyze their distribution between the different regions of fish oil-in-water emulsions, reporting the eventual antagonist and/or synergistic effects between them. Hopefully, these studies will identify potential interactions between antioxidants and will contribute to both understanding these cooperative effects and to improving the antioxidant efficiency of *Bryophyllum* plant extracts in preventing lipid oxidation. Furthermore, *Bryophyllum* extracts may constitute a source of bioactive compounds with antioxidant activity that may be of particular interest in additional sectors, such as the cosmetic and pharmaceutical industries.

## Figures and Tables

**Table 1:** Total phenolic content (TPC), expressed as total gallic acid equivalents (GAE, mM) of *Bryophyllum* species investigated and their antiradical activity expressed as IC50 value (in µM). GA was included as reference standard. Values were expressed as the mean ± standard deviation of three independent replicates. Different letters indicate significant differences (α = 0.001). BH: *Bryophyllum × houghtonii*; BD: *Bryophyllum daigremontianum*; BT: *Bryophyllum tubiflorum*.

Bryophyllum Extract	GAE (mM)	IC50 (µM)
BH	5.91 ± 0.17 ^a^	239.6 ± 1.7 ^b^
BD	5.13 ± 0.26 ^b^	259.7 ± 0.8 ^c^
BT	3.43 ± 0.81 ^c^	259.5 ± 2.9 ^c^
**Reference Standard**		
GA	-	163.4 ± 2.4 ^a^

## Supplementary Materials

The following are available online at https://www.mdpi.com/2223-7747/9/8/1012/s1. **Table S1:** Preliminary identification of phenolic compounds found in *Bryophyllum* plant extracts, together with mass spectral data, [M-H]- (m/z) and structure.
