# Supplementary material for: Exploring the Use of Bryophyllum as Natural Source of Bioactive Compounds with Antioxidant Activity to Prevent Lipid Oxidation of Fish Oil-In-Water Emulsions
Source: Plants (Basel). 2020 Aug 11;9(8):1012. doi: 10.3390/plants9081012 (PMC7464648; doi:10.3390/plants9081012)
Supplement: Supplementary file 1 [file plants-09-01012-s001.pdf]

Table 1. Preliminary identification of phenolic compounds found in *Bryophyllum* plant extracts, together with mass spectral data, [M-H]- (m/z), and structure.

Compound.	$[M\text{-}H]^-$ (m/z)	Structure
Ferulic acid	355	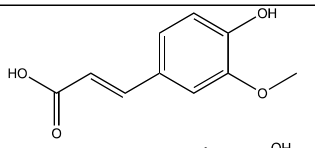
Coumaric acid	325	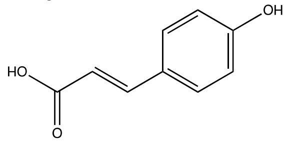
Myricetin-O-rhamnoside	463	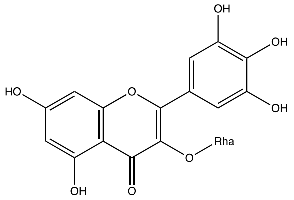
Myricetin-O-rhamnoside-O-hexoside	595	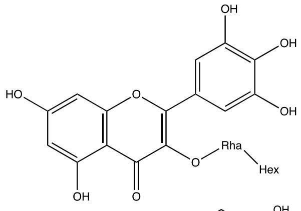
Quercetin-O-hexoside	463	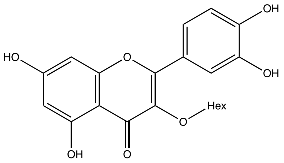
Kaempferol-O-rhamnoside-O-pentoside	563	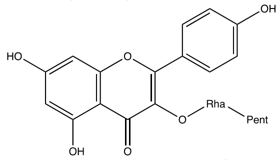
Malvidin-O-hexoside	493	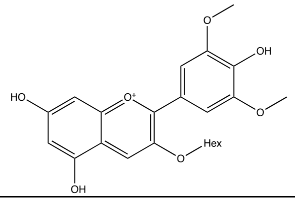
